# Socialistic Theories in Their Medical Aspect

**Published:** 1856-05

**Authors:** 


### EDITORIAL DEPARTMENT.

"*Socialistic Theories in their Medical Aspect.*"—Sometime last fall we ventured, with some misgivings, an article with the title given above, sketching in a cursory manner the intimate connection between the two apparently antipodal subjects, and taking as our text a certain disreputable novel, (put forth as the mixed advocate of water cure and free-love,) called Mary Lyndon.

We are frank to confess that we now and then dip into a novel, with a zest but little less than that of our boyhood, if it is only a book with an object, speaking straight out for some real or fancied reform. We have recently made a period of semi-illness the excuse for an indulgence of this sort, and have read "Alton Locke, Tailor and Poet," a book republished in this country some six years ago. Its author, the Rev. Mr. Kingsley, is a very free-thinking minister of the Church of England.

Some years ago we noticed in another work of his, called "Yeast," (a name typical of the fermenting mind of the day) what we then thought to be merely a curious coincidence in the grouping of his characters, All his "weak sisters," his sentimentalists, and rose-pink reformers, were homœopathists. A similar grouping in "Alton Locke," proves this to have been a really artistic stroke of genius. It is more especially to this that we designed to call attention in the present article; but we cannot refrain from giving speech to the thought that perhaps the medical profession has, in and of itself, a character which harmonizes it with certain elements of popular thought, while it antagonizes to an equal degree with others.

Every man with a heart recognizes the fact that in the relations between rich and poor, the rulers and the governed, the latter class have denied them a certain right which of all others is, or should be, inalienable—the right to health, to free air, pure water, and wholesome food. The mission of sanitary reform is one which belongs to our profession; it should be the teacher, not only of the poor, but of the authorities. It has no higher duty, no purer, more ennobling labor to perform. To a certain extent it has met this duty. Every volume of the Transactions of our great medical congress, or of our state societies, has teemed with proof of the responsibility of man for disease—has pointed out that he who sows corruption must reap fever.

But this is not all our duty. It is easy to be bold, to be eloquent, in general terms among ourselves—it is not so easy to take the field against wealth, and taxation, and the incompetency of demagogue officials; and point out the pestilence breeding at our own doors; to build up enemies among our neighbors by a fearless exposure of wrongs where they exist. But this is what must be done before the noble profession of medicine can wash its hands of the iniquitous murders daily done under our laws and their executors, and take its true position among the reforming agencies of the day.

Outside of the profession men have been found to do this, and to the shame of medical men be it said, the pens of Dickens and Kingsley, and the "People's Songs" of Charles Mackay have done more to the furtherance of sanitary reform in England than all the solemn reports of all the committees appointed by medical societies to search out this evil.

And why? Because they dealt in no prosy scientific generalities; they fixed upon well-known fever haunts, alms-houses, or jails, and *wrote them down as individualities.* Taking some prominent evil, they attacked that and not the system. They wrote strong, nervous articles with strong personal applications; put themselves in peril of libel-suits, arrayed against them official stupidity, red-tape indolence, and wealthy avarice. And if any man conceives that they have won this honest immortality as lovers of their kind, without risk, and threats, and insults, he is grievously mistaken.

Is there no need of such action in this freer, richer, more enlightened country? Every physician who reads this knows of some one evil which it is his duty to attack. Every county society has room for labor in its own poor-house or jail. But we need not travel abroad. Here in this new city, with vacant lots as numerous as its buildings, unscrupulous landlords pack their hundreds of human beings under one narrow roof, to breed a cholera panic for the coming summer. The Board of Health feel compelled, even at this early season, to exert all their powers in this regard. And if there is any one thing in the history of this Journal of which we are proud, it is that in its earlier and feebler days, when it could ill afford enemies, it spoke the manly truth about the old poor-house on Prospect Hill, and worked out the only possible reform—the abandonment of the premises. And yet how soon the evil bred itself again in new quarters. The new poor-house became another bed of pestilence. The lazy indifference of those having it in charge allowed the simplest rules of drainage to be neglected, and poured the waters of every rain beneath the rotting timbers of a crowded house. That was a true Nemesis which made a cholera victim of him whose own neglect in this particular brought on the scourge.

Well. That is done with. It seemed, when the authorities had yielded a feeble sham consent to the stern demand of the people for reform, when they had commenced a system of mock improvements which would have failed of its purpose as a matter of course; as if another Nemesis had punished their stingy policy by the fire which destroyed the edifice, necessitating a new construction on a far better, if not a perfect plan. But the old leaven is not yet exhausted, and it is now as much as ever the duty of the press and the county society to insist upon holding no share in that policy of cheap services, which must inevitably result in disaster to the county and the poor alike.

But we have wandered a long way from "Alton Locke." We proposed to make a few selections. Here is one in which the hero, worn by his daily toil on the tailor's bench and shut out from his miserable home, faints from exhaustion in the streets. He is picked up by a party of medical students "on a spree," who take him to a gin-shop, stimulate him freely, and finally put him to bed, against the strong remonstrance of the publican, who only yields "on being reminded of the value of a medical student's custom!" They sit up all night with him, nursing him as tenderly as they could under the inspiration of brandy. The chapter closes with the following bit of philosophy, which is true the world over.

"Rough diamonds, indeed! Your early life may be a coarse, too often a profligate one—but you know the people, and the people know you; and your tenderness and care, bestowed without hope of repayment, cheers daily many a poor soul in hospital wards and fever cellars—to meet its reward some day at the peoples hands. *Alas! for the society which in after life stifles too many of your better feelings, by making you mere flunkies and parasites, dependent for your livelihood on the caprices and luxuries of the rich.*"

Alton takes his place on the board of a tailor shop, and listens to the following explanation of why he will die six months sooner there than on the first floor:

"Acause you get all the other floors' stinks up here, as well as your own. Concentrated essence of man's flesh, is this here as you 're a-breathing. Cellar work-room we calls Rheumatic Ward, because of the damp. Ground-floor's Fever Ward—them as do n't get dysentery gets typhus—your nose 'd tell yer why if you opened the back windy. First floor's Asthma Ward—

do n't you hear 'um now through the cracks in the boards, a-puffing away like a nest of young locomotives? And this here more august and upper-crust cockloft is Consumptive Hospital. First you begins to cough, then you proceed to expectorate, &c. &c."

We once attempted to describe the precisely similar arrangement of the floors in the former Erie County Poor-House, but it was a failure compared to this graphic sketch.

Further along, Locke quarrels with his editor, and gives him the following piece of invective:

"You a patriot! You are a humbug! Look at those advertisements and deny it if you can. Crying out for education, and helping to debauch the public mind with Voltaire's "Candide" and Eugene Sue — swearing by Jesus, and puffing Atheism and blasphemy — yelling at a quack government, quack law, quack priesthoods, *and then dirtying your fingers with half crowns for advertising Holloway's Ointment, and Parr's Life Pills.* You are simply a humbug, a hypocrite, and a scoundrel; and so I bid you good morning."

The passage shows something in what light the patent-medicine swindle is regarded among the better class of English mind. The "Times," the great impersonation of the power of the press, refuses to publish any advertisement of this sort. When shall we have an equally good *morale* on this side the water?

